# SSH1 expression is associated with gastric cancer progression and predicts a poor prognosis

**DOI:** 10.1186/s12876-018-0739-5

**Published:** 2018-01-16

**Authors:** Yusufu Maimaiti, Maimaitiaili Maimaitiming, Yiliang Li, Saifuding Aibibula, Azatijiang Ainiwaer, Aikebaier Aili, Zhenzhu Sun, Kelimu Abudureyimu

**Affiliations:** 1 Department of General Surgery (Research Institute of Minimally Invasive), People’s Hospital of Xinjiang Uygur Autonomous Region, Urumqi, 830000 China; 2 Department of Pathology, People’s Hospital of Xinjiang Uygur Autonomous Region, Urumqi, 830000 China

**Keywords:** SSH1, Gastric cancer, Progression, Survival analysis

## Abstract

**Background:**

Slingshot homolog-1 (SSH1) plays an important role in pathological processes, including in the occurrence and development of tumours. The purpose of this study was to determine whether SSH1 is a key biomarker with prognostic value for survival in patients with gastric cancer.

**Methods:**

We performed immunohistochemistry (IHC) on tissue microarrays containing 100 gastric cancer specimens to evaluate SSH1 protein expression. The association of pathological characteristics with cumulative survival was determined by Kaplan-Meier analysis. A Cox proportional hazards model was generated in the multi-factorial survival analysis to identify univariate prognostic factors of GC.

**Results:**

SSH1 expression level in gastric cancer tissues was significantly associated with lymph node metastasis (*P* = 0.032). Additionally, multivariate regression analysis clearly indicated that SSH1 expression was significantly correlated with poor clinical outcomes of patients with gastric cancer (*P* = 0.016). Multivariate analyses showed that SSH1 was the best predictor of poor prognosis in patients with gastric cancer (*P* = 0.030).

**Conclusions:**

SSH1 expression is associated with gastric cancer progression and predicts a poor prognosis. SSH1 may play an important role in the development of gastric cancer, and it is a promising target for prevention and/or treatment of gastric cancer.

## Background

Gastric cancer (GC) is a common type of malignancy and the second leading cause of cancer-related death in China [1]. Unfortunately, the incidence of GC has been increasing in recent years; there were 1313 thousand incident cases and 819 thousand deaths in 2015 [2]. Therapeutic approaches to GC include surgical treatment, chemotherapy, radiotherapy, and gene therapy. However, the prognosis for GC patients remains poor [3],patients with early-stage GC have nonspecific symptoms, and lymph node invasion or distant metastasis has already occurred by the time they are diagnosed [4]. Therefore, it is important to identify biomarkers that are related to early diagnosis and prognosis.

Cancer cell migration and invasion lead to GC metastasis, which accounts for most cancer-related deaths. The metastasis of GC is a complex, multistep pathological process that involves several signalling pathways. Emerging evidence has shown that the balance between protein phosphorylation and dephosphorylation is crucial in the regulation of cell signalling, and failure to maintain an appropriate balance is known to play a critical role in cancer development [4].

In mammalian cells, SSH phosphatases are encoded by three genes (SSH1, SSH2, and SSH3) that encode members of a family of serine/threonine protein kinases that dephosphorylate the phospho-serine residue of cofilin [5, 6] and reactivate cofilin through phosphorylation at serine-3 [7]. SSH1 was found to be overexpressed in pancreatic cancer (PC) and to contribute to tumour cell migration [8]. Recent studies have demonstrated that SSH1 is a key regulator of vascular smooth muscle cell migration [9]. When cells are stimulated by growth factors or chemokines, SSH1 accumulates in the F-actin-rich lamellipodium and likely plays a critical role in polarized cell migration by maintaining the local activation of cofilin and the rapid turnover of actin filaments at the leading edge of the migrating cell [10]. In addition, there is evidence suggesting that SSH1 plays an important role in cancer development. However, there is currently no evidence suggesting that SSH1 expression correlates with GC prognosis. Thus, studies are needed to determine whether SSH1 is a prognostic biomarker for GC. We designed a tissue microarray (TMA) covering 100 patients with 8 years of follow up. We performed IHC analysis to investigate the correlation between SSH1 expression and clinico-pathological characteristics and to determine whether SSH1 can serve as a prognostic marker.

## Methods

This study was approved by the hospital ethics committee, and written informed consent was received from the patients. We used a retrospective design to analyse GC tissue samples and all patients with GC had pathologically proven disease and underwent surgical operation between July 2006 and April 2007. All patients were followed up for 19–105 months (average, 49.74 months). The GC tissue and adjacent-to-carcinoma TMAs with a total of 100 cases of GC and 80 cases of adjacent-to-carcinoma paraffin-embedded tissue specimens were purchased from the National Engineering Center for Bio Chips, Shanghai, People’s Republic of China. Complete histological data for the patients with GC were available.

**Fig. 1:**
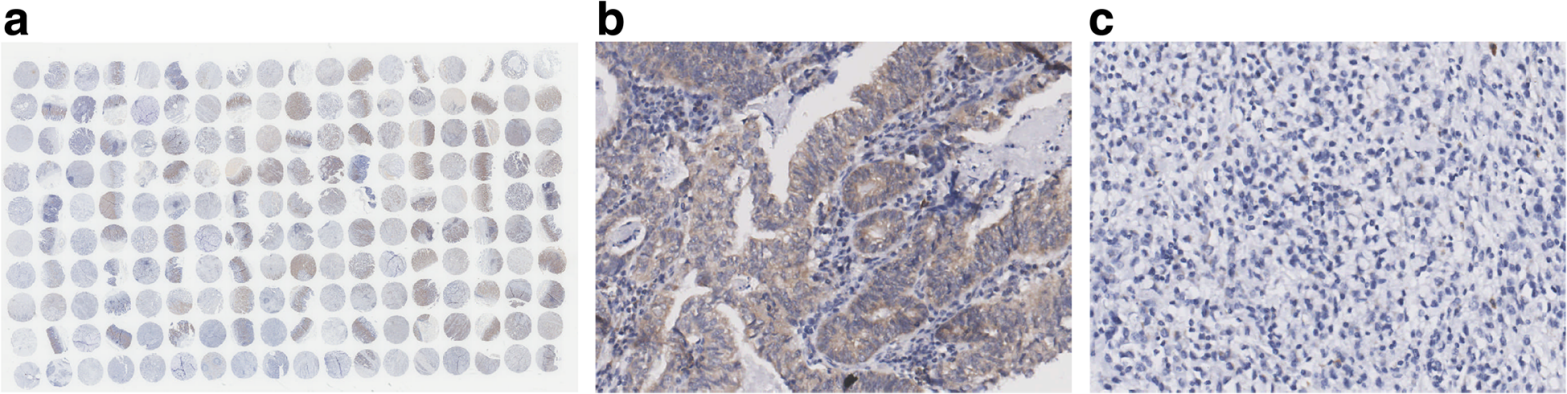
Tissue microarray and IHC staining of SSH1 in GC tissues. **a** Tissue microarray (100 cases of GC tissue and 80 cases of adjacent-to-carcinoma tissue), 1× magnification. **b** Positive expression of SSH1 in cancer tissues; 200× magnification. **c** Negative expression of SSH1 in cancer tissues; 200× magnification

The patients’ clinical characteristics, such as gender, age, tumour location, tumour size, pathological type, pathological grading, tumour infiltration, lymph node metastasis, total lymph nodes dissected, total number of positive lymph nodes (pathology), and distant metastasis, were obtained from their medical records (Table 1). Metastasis status was expressed using the pathologic stage of the disease determined according to the seventh edition of the American Joint Committee on Cancer (AJCC)/International Union Against Cancer TNM classification system. TMA and IHC staining of SSH1 in GC tissue (100 cases) and adjacent-to-carcinoma tissue (80 cases) was performed in our study (Fig. 1a).

**Table 1:** Clinico-pathological features of the 100 patients with GC

Clinico-pathologic features	Cancer	Adjacent to carcinoma
SSH1 (+)	66	66
SSH1 (−)	34	14
Age (years)		
≤ 55	21	17
>55	79	63
Gender		
Male	64	47
Female	36	33
Location		
Cardiac	11	10
Fundus	8	8
Corpora	18	13
Sinuses	57	46
Total	6	3
Pathological type		
Glandular	64	50
Signet ring cell	12	9
Mucinous cell	11	9
Undifferentiation	12	11
Missing	1	1
Lymph node metastasis		
Positive	79	63
Negative	21	17
Tumour infiltration		
T1	6	5
T2	7	4
T3	66	55
T4	20	15
Missing	1	1
Size		
≥ 5 cm	59	45
<5 cm	39	35
Missing	2	0
AJCC staging		
Stage I	10	7
Stage II	33	29
Stage III	49	36
Stage IV	7	7
Missing	1	1

### IHC and scoring

Immunohistochemistry (IHC) staining was used to detect SSH1 expression in the GC and adjacent-to-carcinoma TMA (Fig. 1b & c). To evaluate SSH1 expression, we used a SSH1-specific antibody from Abcam (ab76943; Cambridge, UK) at a dilution of 1:1000, based on the manufacturer’s protocol. Two experienced pathologists who were blinded to the patients’ clinical information independently evaluated and recorded the IHC results. Brown-yellow staining indicated SSH1-positive expression. According to the staining intensity and percentage of positive cells, the tissue samples were comprehensively scored. The SSH1 staining intensity in the tumour cells was graded as 0–3, and the percentage of SSH1-positive cells was graded according to the proportion of positive cells using a 0–4 grading scale (0: 0%–5%; 1: 6%–25%; 2: 26%–50%; 3: 51%–75%; 4: 76%–100%). The final SSH1 expression score (positive or negative) was calculated as the sum of both grades (negative: total grade = 0–3; positive: total grade = 4–7).

### Survival and statistical analysis

#### Methods

All slides were scanned using an Aperio Scan Scope slide scanner, and images of representative areas were obtained using Image Scope software (Aperio) and Adobe Illustrator. The primary clinical and histopathological data were compiled using EpiData software (version 3.1; EpiData Association, Odense, Denmark).

#### Statistical analysis

The Chi squared test and Fisher’s exact probability method were adopted to compare SSH1 expression levels and clinico-pathological parameters in the 100 patients with GC. The Kaplan-Meier survival curve method and log-rank test were applied to compare the survival rate between groups. A Cox proportional hazards model was generated in the multi-factorial survival analysis to identify univariate prognostic factors. SPSS software (version 22.0; SPSS Inc., Chicago, IL, USA) was used to analyse the data. All tests were two-sided, and *P* values <0.05 indicated statistical significance.

## Results

The detailed clinico-pathological characteristics of these 100 patients are provided in Table 1. According to the total lymph nodes dissected and positive lymph nodes results, which were confirmed by pathology, we calculated the positive lymph node ratio, which defined as ratio of positive lymph nodes to all lymph nodes removed, is a powerful prognostic factor in cancer [11], and identified 43 cases with a positive lymph node ratio ≥ 0.5 and 57 cases with a positive lymph node ratio < 0.5. Tumour infiltration was at T1 in 8 cases, T2 in 7 cases, and T3 in 64 cases and T4 in 20 cases; 1 case was unknown. Lymph node metastasis (LNM) was positive in 79 cases, and 21 cases had no LNM. Regarding pathological grade (I-IV), there were 15 cases of grade II, 74 cases of grade III (II-III, III), and 11 cases of grade IV (III-IV). According to the seventh edition of the AJCC staging system, 10 cases were phase I, 33 were phase II, 49 were phase III, 7 were phase IV, and 1 was unknown.

### Association between SSH1 expression in GC and adjacent-to-carcinoma tissues and clinico-pathological features of GC

In our study, 66 cases of GC tumour tissue (66%) and 66 cases of adjacent-to-carcinoma tissue (82.5%) showed positive SSH1 expression, indicating that SSH1 is expressed at a high frequency. The difference in SSH1 expression between GC and adjacent-to-carcinoma tissues was not statistically significant (*P* = 0.769; Table 2). We investigated the relationship between SSH1 expression levels in GC and adjacent-to-carcinoma tissues and clinico-pathological features of GC (Table 3), and no significant correlation was found between SSH1 expression in GC tissue and sex, age, tumour size, tumour location, pathological type, pathological grade, positive lymph node ratio or AJCC stage (all *P* > 0.05, Table 3); there was a correlation with only lymphatic metastasis (*P* = 0.032; Table 3). Additionally, there was no significant difference between SSH1 expression in adjacent-to-carcinoma tissues and clinico-pathological features of GC, including age, sex, tumour location, tumour size, pathological type, pathological grade, lymphatic metastasis and AJCC stage.

**Table 2:** Relationship between cancer tissues and adjacent-to-carcinoma tissues

		Cancer		Correlation	*P*-value
		SSH1(+)	SSH1(−)		
Adjacent to Carcinoma	SSH1(+)	25	41	−0.039	0.769
	SSH1(−)	6	8

**Table 3:** Association between SSH1 expression in GC and adjacent-to-carcinoma tissues and clinico-pathological features of GC

Variables	Cancer tissue			Adjacent-to-carcinoma tissue		
	SSH1(+)	SSH1(−)	*P-*value	SSH1(+)	SSH1(−)	*P-*value
Age (years)			0.311			0.722
≤ 55	5	16		15	2	
>55	29	50		51	12	
Gender			0.098			0.555
Male	18	46		40	7	
Female	16	20		26	7	
Location			0.201			0.112
Sinuses	16	41		23	8	
Other	18	25		20	6	
Lymph node metastasis			0.032*			0.461
Positive	31	48		53	10	
Negative	3	18		13	4	
Positive lymph node ratio			0.201			0.112
≥ 0.5	18	25		29	6	
<0.5	16	41		37	8	
Tumour infiltration			0.893			0.518
T1	2	6		6	0	
T2	2	5		2	3	
T3	23	41		43	10	
T4	6	15		14	2	
Pathological grading			0.285			0.538
2	4	11		11	2	
3	24	50		48	9	
4	6	5		7	3	
Size			0.181			0.137
≥ 5 cm	24	35		40	5	
<5 cm	10	29		26	9	
AJCC staging			0.189			0.676
Stage I	3	7		5	2	
Stage II	9	24		25	4	
Stage III	18	31		30	6	
Stage IV	4	3		6	1	

### Survival analysis

**Fig. 2:**
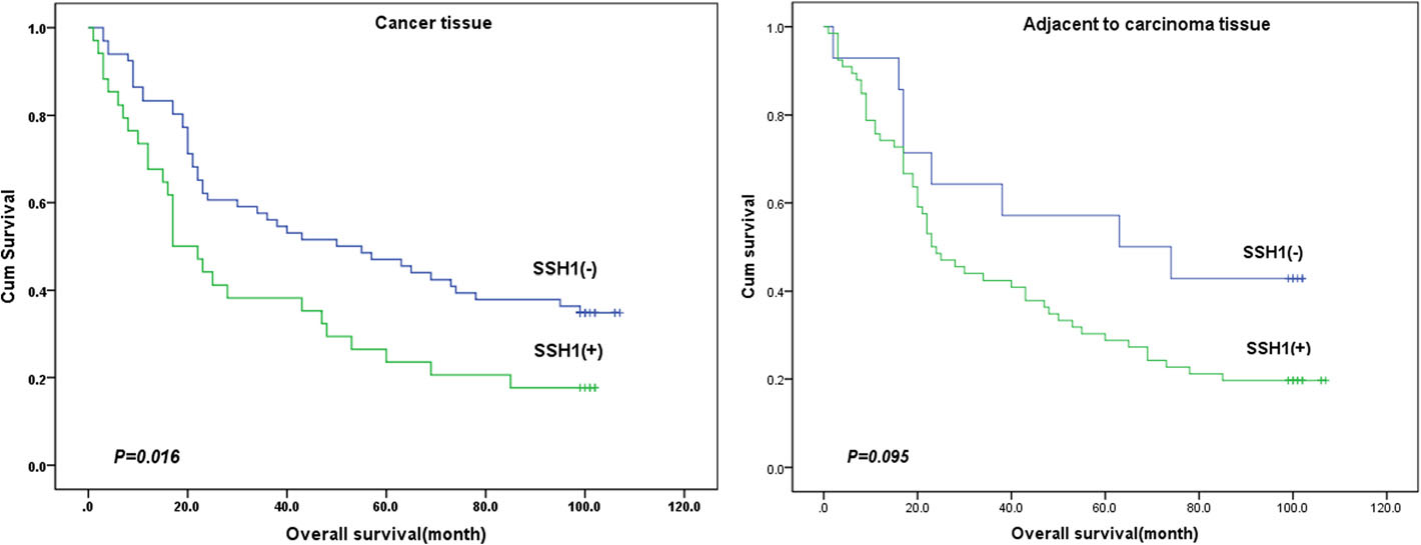
Kaplan-Meier curves show that the survival time of patients with SSH1-positive GC and adjacent-to-carcinoma tissues correlated with prognosis

Survival analysis showed that the median survival time of the 100 patients with GC was 39 months. Kaplan-Meier survival analysis demonstrated that the survival time in patients with SSH1-positive GC tissues was significantly correlated with prognosis (*P =* 0.016; Fig. 2**)**. However, SSH1 expression in adjacent-to-carcinoma tissues was not significantly correlated with the prognosis of patients with GC in our study (*P =* 0.095, Fig. 2). Moreover, we found that some clinico-pathological parameters, including lymph node metastasis, positive lymph node ratio, pathological grading, tumour size, AJCC staging and tumour infiltration (*P =* 0.024, *P < 0*.001, *P =* 0.004, *P* = 0.004, *P <* 0.001, *P =* 0.003, respectively; Table 4), were significantly related to prognosis, while gender, age, tumour location and pathological type were not correlated with the prognosis of patients with GC (all *P* > 0.05; data not shown).

**Table 4:** Univariate and multivariate analyses of overall survival of GC patients

Variables	Univariate analysis			Multivariate analysis		
	HR	95% CI	*P-*value	HR	95% CI	*P-*value
Age (years)	1.043	0.75–1.132	0.288			
Gender	1.142	0.952–1.378	0.588			
Tumour Location	1.024	0.944–1.389	0.52			
Lymph node metastasis	1.336	1.045–1.092	0.024*	1.522	0.452–5.124	0.472
Positive lymph node ratio	2.584	1.267–7.321	<0.001*	2.612	1.214–5.622	0.014*
Pathological type	1.018	0.694–1.258	0.474			
Pathological grade	2.094	1.316–4.167	0.004*	0.474	0.153–1.464	0.194
Tumour size	1.921	1.156–3.126	0.004*	0.367	0.049–2.752	0.329
Tumour infiltration	1.321	1.054–2.421	0.003*	1.524	0.953–2.439	0.079
AJCC staging	2.126	1.335–6.075	<0.001*	1.615	0.735–3.547	0.233
Adjacent-to-GC tissue-SSH1	1.164	0.925–1.637	0.095			
GC tissue-SSH1	1.564	1.109–2.352	0.016*	1.787	1.056–3.021	0.030*

The univariate analysis model showed that pathological grade, positive lymph node ratio, size, AJCC stage and SSH1 expression in cancer tissue were important factors for the predication of prognosis in patients with GC. All the variables that were statistically correlated with overall survival in the univariate analysis were included in the multivariate regression analysis. The results showed that positive lymph node ratio and SSH1 expression (*P* = 0.014 and *P* = 0.030, respectively) were prognostic factors influencing the survival of patients with GC (Table 4). These data clearly indicated that SSH1 expression is an exceptional predictor of poor prognosis for GC.

## Discussion

Tumour development is a complex process, and multiple factors and steps participate in tumour malignant behaviour. The actin cytoskeleton plays important roles in cell migration, affecting such processes as cell-substrate adhesion, protrusion, phagocytosis and cytokinesis [12], and actin proteins form a large family and are central players in cell shape and movement [13]. SSH1-mediated cofilin activation is an essential regulator of actin dynamics [14]; SSH1 and cofilin are highly expressed or highly activated in various malignant tumours, increasing the rate of invasion and metastasis of tumour cells through different mechanisms and pathways [15, 16]. However, there is currently little evidence suggesting that SSH1 expression correlates with GC prognosis. The results of this study showed that SSH1 expression in GC tissues predicted GC lymph node metastasis based on TNM stage (*P* = 0.032; Table 3) and poor survival (*P* = 0.016; Table 4 & Fig. 2). SSH1 expression is indeed a useful prognostic factor for GC and is significantly associated with a poor prognosis.

SSH1 dephosphorylates and activates cofilin, which is a member of the actin-depolymerizing factor/cofilin family of proteins [12, 17] that regulates formation of the actin cytoskeleton. Tumour invasion and metastasis, the main causes of cancer-related death, are directly associated with cofilin activity [18, 19]. Yusufu et al. [20] reported that the cofilin phosphorylation status plays a critical role in breast cancer, and dephosphorylated cofilin is related to lower overall survival. It has been reported that cofilin is related to GC progression [21]. Wang et al. [8] suggested that SSH1 may be a potential target to prevent or treat PC invasion and metastasis by regulating cofilin phosphorylation. Combined with our results, we speculated that SSH1 is a key protein that associates with F-actin, which plays a key role in the malignant biological behaviour of GC cells.

According to our IHC analysis and microarray data, SSH1 expression was not different between GC and adjacent-to-carcinoma tissues (*P* = 0.769; Table 2). However, in this study, SSH1 was expressed in GC tissue, and its expression level was positively correlated with tumour LNM and poor prognosis; multivariate regression analysis clearly indicated that SSH1 expression in GC tissues was an exceptional predictor of poor prognosis (*P* = 0.030; Table 4). SSH1 affects tumour migration by altering cofilin activity, and researchers have proposed studying this interaction to gain better insight into the pathogenesis of digestive system tumours. Wang et al. [8] designed a study to explore the role of SSH1 in the development of PC; their results showed that SSH1 overexpression is related to cofilin activity and contributes to LNM and tumour cell migration, findings that were completely consistent with our results.

Not a single gene was activated within the migrating and invading tumour cells. Tumour invasion and metastasis, the main cause of cancer-related death, is directly associated with cofilin activity [18, 22]. Tan [16] et al. reported that the poor prognosis of patients with colorectal cancer is related to regulation of F-actin polymerization through the SSH/cofilin pathway. The function of the SSH/cofilin signalling pathway in actin polymerization and dynamics was studied using small interfering RNA (siRNA) silencing of SSH1,down-regulation SSH1 led to decreases in vascular cell migration and metastatic progression [9, 23]. Cell migration is associated with pathological remodelling at the site of vascular injury and with the invasive capacity of cancer cells. Our results showed that SSH1 expression level in GC was related to poor patient survival (*P* = 0.016). Therefore, SSH1 is the most important factor in cancer cell migration, and the abovementioned findings may indicate why the regulation of SSH1 activity is a promising therapeutic strategy to prolong the survival of cancer patients. As a regulator of cofilin, SSH1 may be an important regulatory factor in GC cell invasion and metastasis and may be closely related to the occurrence and development of GC. Therefore, the regulation of SSH1 activity is a promising therapeutic strategy for GC.

At present, there are three important limitations to our study. First, we used a retrospective design to analyse GC tissue samples from patients treated at a single centre and selection bias was unavoidable. Second, we analysed only the correlation with overall survival and could not access data regarding recurrence-free and disease-free survival. Third, our study did not include any molecular experiments.

## Conclusion

In summary, this study clarified the clinical significance of SSH1 expression level in GC patients and showed that SSH1 activation plays an important role in GC progression and prognosis. Importantly, SSH1 dephosphorylates and activates cofilin, and SSH1 may play an important role in cell migration and cancer development through SSH/cofilin pathways. Our study indicated that SSH1 expression is a promising biomarker for the prevention and/or treatment of GC.

## Highlights

1. Gastric cancer (GC) is a common type of malignancy and the second leading cause of cancer-related death in China [1]. Unfortunately, the prognosis for GC patients remains poor,therefore, it is important to identify biomarkers that are related to early diagnosis and prognosis.
2. Slingshot homolog-1 (SSH1) plays an important role in pathological processes, including in the occurrence and development of tumours [2–4] . However, there is currently no evidence suggesting that SSH1 expression correlates with GC prognosis. Thus, studies are needed to determine whether SSH1 is a prognostic biomarker for GC.
3. SSH1 is the most important factor in cancer cell migration, in our study clarified the clinical significance of SSH1 expression level in GC patients and showed that SSH1 activation plays an important role in GC progression and prognosis. As a regulator of cofilin, SSH1 may be an important regulatory factor in GC cell invasion and metastasis and may be closely related to the occurrence and development of GC.

## Abbreviations

AJCC: American Joint Committee on Cancer
CI: confidence interval
GC: Gastric cancer
HR: hazard ratio
IHC: immunohistochemistry
PC: pancreatic cancer
SSH1: Slingshot homolog-1
TMA: tissue microarray
